# Impact of age and comorbid heart failure on the utility of smart voice-assistant devices

**DOI:** 10.1093/ehjdh/ztae012

**Published:** 2024-02-16

**Authors:** Pedro Marques, Anahita Emami, Guang Zhang, Renato D Lopes, Amir Razaghizad, Robert Avram, Abhinav Sharma

**Affiliations:** Division of Cardiology, McGill University Health Centre, McGill University, 1001 Decarie Blvd, Montreal H4A 3J1, Quebec, Canada; DREAM-CV Lab, Centre for Outcomes Research, McGill University, 1001 Decarie Blvd, Montreal H4A 3J1, Quebec, Canada; Division of Cardiology, McGill University Health Centre, McGill University, 1001 Decarie Blvd, Montreal H4A 3J1, Quebec, Canada; Division of Cardiology, McGill University Health Centre, McGill University, 1001 Decarie Blvd, Montreal H4A 3J1, Quebec, Canada; DREAM-CV Lab, Centre for Outcomes Research, McGill University, 1001 Decarie Blvd, Montreal H4A 3J1, Quebec, Canada; Duke Clinical Research Institute, Duke University, Durham, NC, USA; Division of Cardiology, McGill University Health Centre, McGill University, 1001 Decarie Blvd, Montreal H4A 3J1, Quebec, Canada; Montreal Heart Institute, University of Montreal, Montreal, Quebec, Canada; Heartwise AI Lab, Montreal Heart Institute Research Center, Montreal, Quebec, Canada; Division of Cardiology, McGill University Health Centre, McGill University, 1001 Decarie Blvd, Montreal H4A 3J1, Quebec, Canada; DREAM-CV Lab, Centre for Outcomes Research, McGill University, 1001 Decarie Blvd, Montreal H4A 3J1, Quebec, Canada

**Keywords:** Amazon Alexa, Voice-assistant device, Heart failure, Age

## Abstract

**Aims:**

The accuracy of voice-assisted technologies, such as Amazon Alexa, to collect data in patients who are older or have heart failure (HF) is unknown. The aim of this study is to analyse the impact of increasing age and comorbid HF, when compared with younger participants and caregivers, and how these different subgroups classify their experience using a voice-assistant device, for screening purposes.

**Methods and results:**

Subgroup analysis (HF vs. caregivers and younger vs. older participants) of the VOICE-COVID-II trial, a randomized controlled study where participants were assigned with subsequent crossover to receive a SARS-CoV2 screening questionnaire by Amazon Alexa or a healthcare personnel. Overall concordance between the two methods was compared using unweighted kappa scores and percentage of agreement. From the 52 participants included, the median age was 51 (34–65) years and 21 (40%) were HF patients. The HF subgroup showed a significantly lower percentage of agreement compared with caregivers (95% vs. 99%, *P* = 0.03), and both the HF and older subgroups tended to have lower unweighted kappa scores than their counterparts. In a post-screening survey, both the HF and older subgroups were less acquainted and found the voice-assistant device more difficult to use compared with caregivers and younger individuals.

**Conclusion:**

This subgroup analysis highlights important differences in the performance of a voice-assistant–based technology in an older and comorbid HF population. Younger individuals and caregivers, serving as facilitators, have the potential to bridge the gap and enhance the integration of these technologies into clinical practice.

**Study Registration:**

ClinicalTrials.gov Identifier: NCT04508972.

## Graphical Abstract

**Graphical Abstract:**
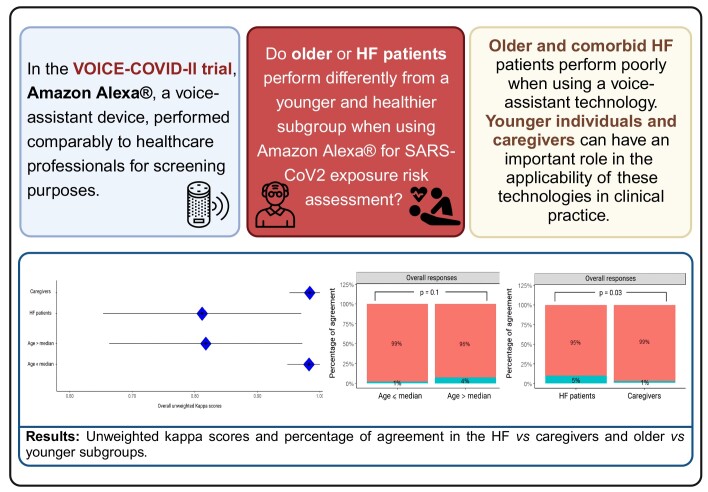
HF, heart failure.

## Introduction

The rising prevalence of cardiovascular diseases, such as heart failure (HF), is causing substantial pressure on healthcare systems. The COVID-19 pandemic has further strained healthcare resources and led to the accelerated proliferation of artificial intelligence-based systems.^1^ Artificial intelligence-based technologies can potentially be integrated into clinical practice to streamline care in older and HF patients.^2^ Smart voice-assistant systems performed comparably with healthcare professionals for screening purposes in a HF population and caregivers.^3^ Whether older or HF patients would perform differently from a younger and healthier subgroup and to what extent that can challenge their application in clinical practice is unclear. The VOICE-COVID-I9-II trial (NCT04508972) was one of the first randomized trials to evaluate the utility of a voice-based system (Amazon Alexa) to screen both patients with HF and caregivers for SARS-CoV-2 exposure risk.^3^ With these data, we aim to analyse the impact of increasing age and comorbid HF, when compared with younger participants and caregivers, and how these different subgroups classify their experience using an a voice-assistant device, for screening purposes.

## Methods

The VOICE-COVID-19-II study was a single-centre, open-label, non-interventional, crossover, randomized controlled trial comparing the concordance of a SARS-CoV-2 screening data capture by Alexa to a research coordinator. The trial protocol and main results were previously published.^3,4^ Briefly, HF patients and caregivers from a HF clinic were randomized to a 5-binary COVID-19 screening questionnaire by Alexa or a research coordinator. Excluding the first question on language preference (English vs. French), the remainder were yes/no questions related to COVID-19 exposure risk. Participants subsequently cross over to be screened by the alternate method. After completing the screening questionnaire, all participants agreed to answer an 11-question survey to assess perceived user preferences and Alexa software application engagement, including questions on participant’s comfort, perception relative to data security, and effectiveness of using voice-assistant devices.

The sample size was powered to test the level of concordance between Alexa and the research coordinator in the overall patient population using the percentage of agreement and unweighted Kappa scores. Unweighted Kappa scores were computed using the R package ‘vcd’ and function ‘Kappa’, by subtracting the expected agreement (Pe) to the observed agreement (Po) and dividing this value by the non-expected agreement (1-Pe). In this pre-specified exploratory sub-analysis, we present the level of concordance of HF vs. non-HF (caregivers) and younger vs. older participants included in the trial. The age subgroup was defined as below and above the median age of our sample. For both the percentage of agreement and unweighted kappa scores, age as a continuous variable and HF group were explored in linear regression models, with/without interaction variables and covariates in the univariable and multivariable models. All analyses considered an alpha of 0.05. Data were analysed using R (version 4.2.3). This study was approved by the local Research Ethics Board (2020-6583).

## Results

From the 52 participants recruited with a median age of 51 (interquartile range 34–65) years, 21 (40%) were HF patients and the remainder were caregivers, 36 (69%) were male, and 36 (69%) were English speaking. For the HF subgroup, 81% were male, the median age was 64 (54–71) years, and 10 (48%) had ischaemic cardiomyopathy as the main aetiology (*Table 1*).

**Table 1:** Baseline characteristics for the overall population, the heart failure, caregiver, and older/younger subgroups

Characteristic	Overall	HF subgroup	Caregiver subgroup	Older subgroup	Younger subgroup
Number	52	21	31	26	26
**Randomization order**					
Alexa – research coordinator	26 (50%)	12 (57%)	14 (45%)	12 (46%)	14 (54%)
Research coordinator – Alexa	26 (50%)	9 (43%)	17 (55%)	14 (54%)	12 (46%)
Male sex	36 (69%)	17 (81%)	19 (61%)	22 (85%)	14 (54%)
Age (years), median (IQR)	51 (34–65)	64 (54–71)	38 (28–54)	66 (60–72)	33 (27–39)
**Language**					
English	36 (69%)	13 (62%)	23 (74%)	13 (50%)	23 (88%)
French	16 (31%)	8 (38%)	8 (26%)	13 (50%)	3 (12%)
**Comorbidities**					
Hypertension	10 (19%)	9 (43%)	1 (3%)	10 (38%)	0
Diabetes mellitus	10 (19%)	9 (43%)	1 (3%)	8 (31%)	2 (8%)
Dyslipidaemia	13 (25%)	12 (57%)	1 (3%)	11 (42%)	2 (8%)
Chronic pulmonary obstructive disease	3 (6%)	2 (10%)	1 (3%)	3 (12%)	0
CKD	7 (13%)	6 (29%)	1 (3%)	7 (27%)	0
Anaemia	3 (6%)	3 (14%)	0	2 (8%)	1 (4%)

CKD, chronic kidney disease; HF, heart failure; IQR, interquartile range.

**Figure 1:**
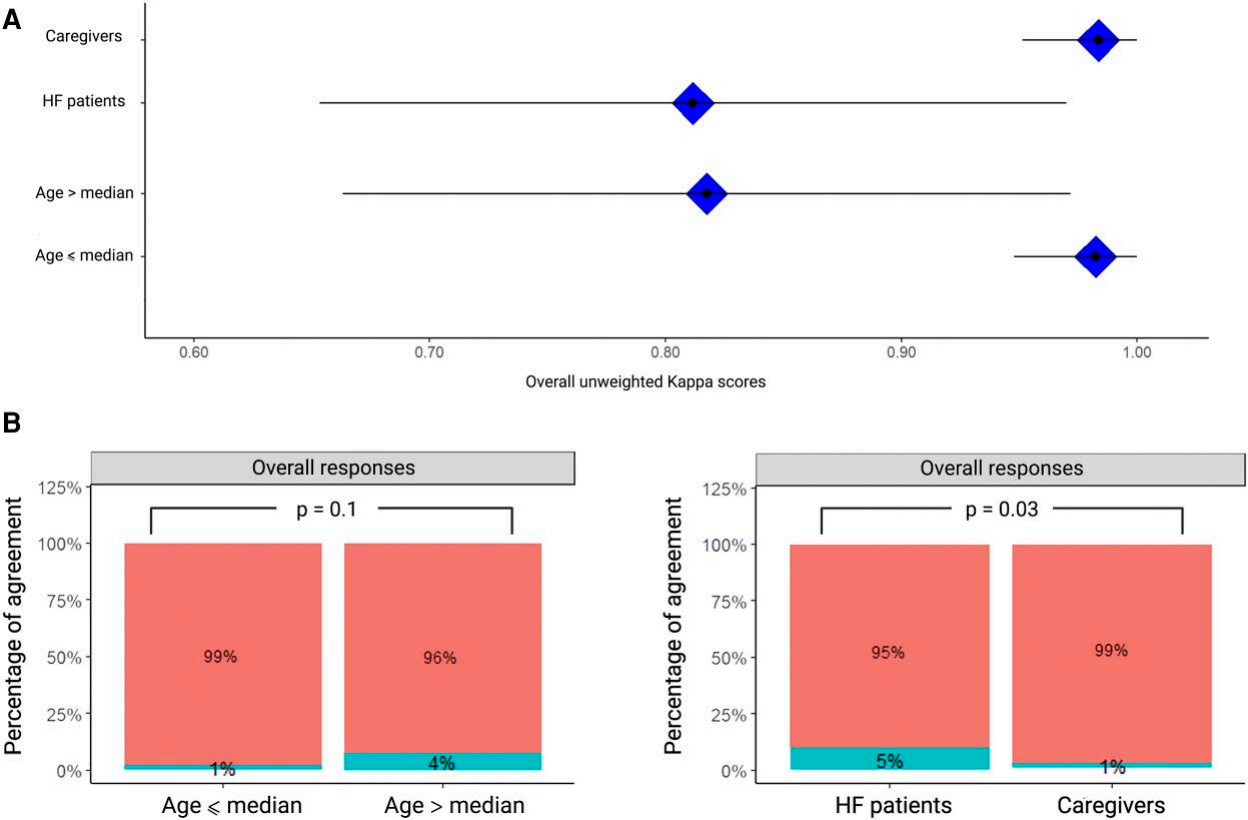
Subgroup analysis of heart failure vs. caregivers and younger vs. older subgroups for the overall responses to the screening questionnaire. (*A*) Unweighted Kappa scores; (*B*) overall percentage of agreement. Level of concordance was evaluated using the unweighted kappa scores (*A*) and the Wilcoxon signed-rank test (*B*).

The results from the HF vs. caregivers and older vs. younger subgroups are illustrated in *Figure 1*. The HF subgroup showed a significant lower percentage of agreement compared with caregivers (95% vs. 99%, *P* = 0.03), while both the HF and the older subgroup tended to have lower unweighted kappa scores than their counterparts (HF vs. caregivers: 0.81, 95% confidence interval (CI) 0.65–0.97 vs. 0.98, 95% CI 0.95–1.00; older vs. younger subgroup: 0.82, 95% CI 0.66–0.97 vs. 0.98, 95% CI 0.95–1.00). In univariable linear regression models, both age and HF status significantly correlate negatively with kappa scores and percentage of agreement. In multivariable linear regression models, age, HF status, and the interaction term also show significant *P*-values (<0.05).

In the post-screening survey, both the HF and older subgroups had less contact with Alexa, found the voice-assistant device more difficult to use, and rated their overall experience poorly than their counterparts (*P* < 0.05, all). No statistical differences were noted in the results between French- and English-speaking participants (*P* > 0.05).

## Discussion

In a standard SARS-CoV2 screening questionnaire, older and HF patients showed lower levels of concordance when using Amazon Alexa, a voice-assistant device, compared with a healthcare professional, than younger participants and caregivers. To our knowledge, this is the first time that a smart voice-assistant device has been evaluated in both patients with HF and caregivers.

There is growing interest in the use of voice-assistant devices to streamline care in chronic conditions, such as HF.^2,5^ Although the main results of our trial showed that Alexa performed comparably with a healthcare professional in the data capture of a standard COVID-19 screening questionnaire, this subgroup analysis shows that older and more comorbid participants clearly show lower rates of overall concordance. In the post-screening survey, the older and comorbid participants classified the screening survey using Alexa more difficult to use compared with younger individuals.

The results from our analysis highlight the differences between these subgroups with regard to data concordance and the potential challenges in the applicability of voice-assistant-based technologies in comorbid HF and older individuals. Heart failure-associated factors as well as lack of familiarity and ease with this type of technologies, as reported in the post-screening survey, can potentially explain the lower concordance seen in the older and more comorbid subgroup. The inclusion of caregivers and younger individuals also pinpoints their potential role to facilitate the widespread use of these technologies.

It should be mentioned that although there are statistically significant differences between subgroups, the small magnitude of difference may not be relevant in clinical practice. Nevertheless, in the univariable and multivariable regression models, both age and HF status significantly correlate with lower overall concordance suggesting that both subgroups affect the concordance of responses and future applications of these technologies into clinical practice should take these potential pitfalls into consideration.

Regarding the results of the post-screening survey, although the older and HF subgroups classified Alexa as less easy to use, there were no differences between subgroups regarding comfort, and no privacy or safety issues were raised. These results suggest that the potential inclusion of these devices into clinical practice would be well accepted by patients.

## Conclusion

In conclusion, this subgroup analysis highlights important differences in the performance of a voice-assistant technology in an older and comorbid HF population. Voice-based technologies have the potential to support and facilitate care delivery. Younger individuals and caregivers can potentially facilitate the applicability of these technologies in clinical practice. Future digital health technologies focused on HF and other related comorbidities must be tested within older populations to ensure optimal utility and comfort with these technologies.

## Lead author biography


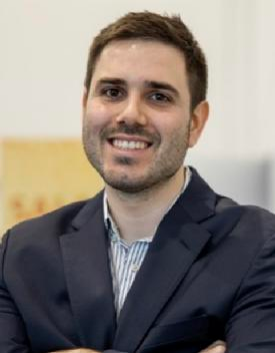
Pedro Marques, MD, is an Internal Medicine Assistant currently working as a Cardiorenal Fellow at the McGill University Health Centre, Montreal, Canada. Dr Marques is also currently enrolled in a PhD programme at the Faculdade de Medicina da Universidade do Porto, Portugal. His current work focuses on multiple aspects of the cardiovascular *continuum*, from cardiovascular risk factors to heart failure. More recently, he has been particularly interested in digital health innovations and their applications to the cardiovascular field.

## Data Availability

The data underlying this article as well as the code for the Alexa device can be made available upon reasonable request to the investigators.
